# The critical role of TRIM protein family in intervertebral disc degeneration: mechanistic insights and therapeutic perspectives

**DOI:** 10.3389/fcell.2025.1525073

**Published:** 2025-02-06

**Authors:** Shangze Li, Wenli Jiang, Fei Chen, Jiao Qian, Jun Yang

**Affiliations:** ^1^ Department of Orthopedics, The Second Affiliated Hospital (Shanghai Changzheng Hospital), Naval Medical University, Shanghai, China; ^2^ Department of Biochemistry and Molecular Biology, College of Basic Medical Sciences, Naval Medical University, Shanghai, China; ^3^ Department of Pharmacy, The First Affiliated Hospital (Shanghai Changhai Hospital), Naval Medical University, Shanghai, China

**Keywords:** intervertebral disc degeneration (IVDD), TRIM protein family, cell death, inflammation, extracellular matrix metabolism

## Abstract

Intervertebral disc degeneration (IVDD) is a leading cause of chronic back pain, contributing significantly to reduced quality of life and global public health burdens. The TRIM (Tripartite Motif-containing) protein family, with its diverse regulatory roles, has emerged as a key player in critical cellular processes such as inflammation, cell death, and extracellular matrix (ECM) metabolism. Recent findings underscore the involvement of TRIM proteins in IVDD pathogenesis, where they regulate stress responses, maintain cellular homeostasis, and influence the functional integrity of nucleus pulposus (NP) and annulus fibrosus (AF) cells. This review explores the multifaceted roles of TRIM proteins in IVDD, highlighting their contributions to pathological pathways and their potential as therapeutic targets. Advancing our understanding of TRIM protein-mediated mechanisms may pave the way for innovative and precise therapeutic strategies to combat IVDD.

## 1 Introduction

Intervertebral disc degeneration (IVDD) is a multifactorial condition driven by complex molecular and cellular mechanisms. Among the numerous factors contributing to IVDD, the tripartite motif-containing (TRIM) protein family has emerged as a significant regulator in processes such as inflammation, apoptosis, and extracellular matrix (ECM) metabolism. This section begins by providing an overview of IVDD and the biological roles of TRIM proteins, establishing the foundation for understanding their mechanistic contributions to IVDD.

### 1.1 Overview of IVDD

IVDD is a multifactorial degenerative disease characterized by the progressive loss of structural integrity and function of the intervertebral disc (IVD). As a leading cause of chronic lower back pain and disability worldwide, IVDD imposes a significant socioeconomic burden (Knezevic et al., 2021). The etiology of IVDD is complex and influenced by genetic predisposition, aging, mechanical overload, and environmental factors (Urits et al., 2019).

The intervertebral disc consists of three main components: the nucleus pulposus (NP), the annulus fibrosus (AF), and the cartilaginous endplates (EPs). The NP provides hydration and compressive resistance, while the AF offers tensile strength and structural support. The EPs serve as a barrier and allow nutrient diffusion between the disc and the adjacent vertebral bodies. In IVDD, these structural components undergo degeneration, leading to reduced hydration of the NP, fibrotic changes in the AF, and calcification of the EPs, ultimately impairing the disc’s biomechanical properties (Knezevic et al., 2021).

At the molecular level, IVDD is characterized by a dysregulated balance between anabolic and catabolic processes within the ECM. Increased expression of matrix-degrading enzymes, such as matrix metalloproteinases (MMPs) and a disintegrin and metalloproteinase with thrombospondin motifs (ADAMTS), leads to ECM breakdown, while decreased synthesis of matrix components such as aggrecan and type II collagen further exacerbates structural deterioration (Hu et al., 2023). In addition, inflammation plays a pivotal role in IVDD pathogenesis. Pro-inflammatory cytokines, including interleukin-1β (IL-1β), tumor necrosis factor-α (TNF-α), and interleukin-6 (IL-6), are upregulated in degenerative discs, promoting ECM degradation, cell death, and neurovascular ingrowth, which contribute to discogenic pain (Chen et al., 2018; Li Wenhao et al., 2022; Cazzanelli and Wuertz-Kozak, 2020).

Another key feature of IVDD is increased cell death, including apoptosis, autophagy dysregulation, and ferroptosis, within the NP and AF. This loss of functional cells compromises the ability of the disc to maintain tissue homeostasis and repair damaged ECM (Tu et al., 2018; Zhang et al., 2021; Lin et al., 2021). Oxidative stress, mitochondrial dysfunction, and endoplasmic reticulum (ER) stress further exacerbate cellular damage, accelerating the degenerative process (Wang et al., 2023; Tang et al., 2019; Hou et al., 2014).

Given its multifactorial nature, the pathogenesis of IVDD remains incompletely understood, and effective therapeutic interventions are limited. Recent advances in molecular biology and regenerative medicine have highlighted potential targets for intervention, particularly in regulating inflammation, mitigating cell death, and restoring ECM balance (Wang et al., 2016; Vo et al., 2016; Adams et al., 2015). These insights provide a foundation for exploring novel therapeutic strategies to halt or reverse the progression of IVDD.

### 1.2 Overview of TRIM proteins

**FIGURE 1:**
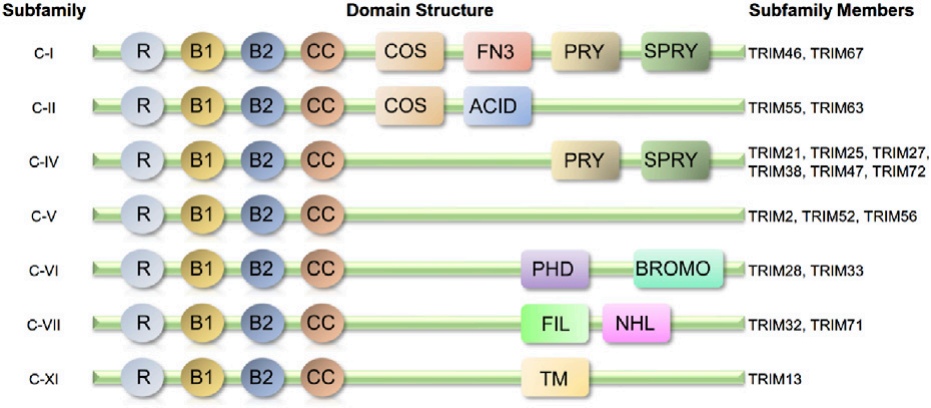
Structural schematic of the TRIM protein family with definite structural data mentioned in this paper (classified by structure). Almost all-related TRIM proteins have a RING-finger domain (R), one or two B-box domains (B) and a coiled-coil domain (CC). ACID, acid-rich region; BROMO, bromodomain; COS, cos-box; FIL, filamin-type I G domain; FN3, fibronection type III repeat; NHL, NCL1, HT2A and LIN41domain; PHD, PHD domain; PRY, PRY domain; SPRY, SPRY domain; TM, transmembrane region.

The TRIM protein family is characterized by its conserved tripartite structure, which includes a RING finger domain, one or two B-box domains, and a coiled-coil domain (Hatakeyama, 2017a). These structural features enable TRIM proteins to function as multifunctional regulators in various biological processes, such as inflammation, autophagy, apoptosis, DNA repair, and protein degradation (Tocchini and Ciosk, 2015). Figure 1. Shows the structural schematic of the TRIM protein family with definite structural data mentioned in this paper (classified by structure).

TRIM proteins play critical roles in maintaining cellular homeostasis and regulating immune responses. They modulate key signaling pathways, such as Nuclear Factor kappa - light - chain - enhancer of activated B cells (NF-κB) and p53, to balance pro-inflammatory and anti-inflammatory activities (Gack et al., 2007; Weng et al., 2014; Hu et al., 2015; Liu et al., 2020). For instance, some TRIM proteins enhance inflammation by stabilizing inflammatory signaling complexes, while others suppress chronic inflammation by promoting the degradation of key signaling molecules (Hu et al., 2015; Marzano et al., 2021). These dual regulatory capabilities highlight the context-dependent nature of TRIM protein functions.

In addition to their role in inflammation, TRIM proteins are essential for cellular adaptation to stress. For example, TRIM32 and TRIM13 promote selective autophagy to clear misfolded proteins and alleviate endoplasmic reticulum (ER) stress (Hatakeyama, 2017a; Lei et al., 2021). In cardiac muscle cells, TRIM55 and TRIM63 regulate proteasomal protein degradation, reducing oxidative damage and preserving cellular integrity (Zhang Jing-Rui et al., 2020).

Moreover, certain TRIM proteins, such as TRIM29 and TRIM32, contribute to genomic stability and apoptosis regulation by mediating DNA repair and activating pro-apoptotic pathways under stress conditions (McAvera and Lisa, 2020; Li et al., 2018). These diverse capabilities suggest that TRIM proteins may act as key modulators in various pathological processes, including IVDD, where inflammation, protein homeostasis, and cell death are central mechanisms (Jiang et al., 2020).

### 1.3 Linking IVDD and TRIM proteins

Recent studies have identified the TRIM protein family as key regulators in the molecular mechanisms of IVDD. TRIM proteins have been shown to influence apoptosis, inflammatory signaling, ECM degradation, and dysregulated autophagy, which are central processes driving IVDD progression (Madhu, Guntur, and Risbud, 2021; Hatakeyama, 2017b; Zhang Litian et al., 2020). These proteins exert their regulatory functions through key pathways such as NF-κB, Mitogen - Activated Protein Kinase (MAPK), and p53, modulating the balance between tissue homeostasis and degeneration. By participating in these interconnected mechanisms, TRIM proteins provide new insights into the complex cellular responses and impaired tissue repair associated with IVDD.

Despite advances in understanding the pathogenesis of IVDD, the role of the TRIM protein family as pivotal regulatory factors remains underexplored. Their unique ability to modulate multiple pathological processes highlights their potential as promising therapeutic targets for IVDD. This review aims to comprehensively analyze the mechanisms through which TRIM proteins influence IVDD pathology and evaluate their therapeutic relevance. A deeper understanding of TRIM-mediated regulatory networks may pave the way for novel, precise therapeutic strategies to manage IVDD in the future.

## 2 Mechanisms underlying IVDD

IVDD is driven by a complex interplay of molecular and cellular mechanisms, which collectively contribute to structural deterioration and functional impairment of the intervertebral disc. Key pathological processes include chronic inflammation, imbalanced ECM metabolism, and increased cell death within NP and AF cells. These mechanisms not only exacerbate disc degeneration but also perpetuate a cycle of cellular dysfunction and tissue damage.

In this section, we examine the central mechanisms underlying IVDD, with a particular focus on the multifaceted regulatory roles of the TRIM protein family. Subsections will explore how TRIM proteins influence inflammation, apoptosis, autophagy, and ECM metabolism, highlighting their potential as therapeutic targets to modulate these pathological processes.

### 2.1 Inflammation in IVDD

The inflammatory response is a major initiating factor and accelerates the progression of IVDD, persisting throughout its entire course. Pro-inflammatory cytokines such as IL-1β and TNF-α activate the NLRP3 inflammasome (NOD-like Receptor Family Pyrin Domain Containing 3), leading to the release of additional cytokines like IL-6 and IL-8. This results in a positive feedback loop that amplifies the inflammatory response, thereby exacerbating IVDD progression (Hu et al., 2023). The activation of NLRP3 increases intracellular calcium levels and oxidative stress, causing NP cell damage and ECM degradation. Studies have shown that ginkgetin, a biflavonoid derived from ginkgo, can alleviate inflammation in IVDD by inhibiting NLRP3 inflammasome activation and reducing pro-inflammatory gene expression in NP cells (Hu et al., 2023). The interplay between inflammation and oxidative stress accelerates NP cell apoptosis and ECM degradation, ultimately creating a vicious cycle that drives the pathological progression of IVDD.

Oxidative stress is a key intrinsic driver of IVDD. With aging, reactive oxygen species (ROS) accumulate in disc cells, while the antioxidant defense system declines in function, resulting in ineffective ROS neutralization and subsequent cellular damage (Chen Fei et al., 2024; Prajapati et al., 2020). ROS directly damage biomolecules such as DNA, proteins, and lipids, activating oxidative stress-related signaling pathways like NF-κB and Keap1-Nrf2, which trigger inflammatory responses and promote cell apoptosis, thereby accelerating IVDD onset (Wang et al., 2023). Nrf2 plays a crucial role in protecting cells from oxidative damage by activating antioxidant genes and promoting autophagy processes (Tang et al., 2019). Antioxidant therapies that reduce ROS accumulation can alleviate oxidative stress-induced disc damage, thereby slowing the progression of IVDD (Hou et al., 2014).

### 2.2 Cell death in IVDD

#### 2.2.1 Apoptosis and ferroptosis in IVDD

Apoptosis, a form of programmed cell death, is a hallmark in the early stages of IVDD, particularly in NP cells. Inflammatory factors like TNF-α and oxidative stress increase apoptosis rates, reducing NP cell numbers and impairing the intervertebral disc’s structural integrity (Chen et al., 2018). Additionally, ferroptosis, an iron-dependent process of lipid peroxidation, contributes to increased ROS levels and exacerbates NP cell damage (Chen et al., 2018; Chai et al., 2019).

For instance, ATF3 (Activating Transcription Factor 3) plays a pivotal role in ferroptosis. Studies reveal that silencing ATF3 can mitigate oxidative stress-induced ferroptosis, thus slowing IVDD progression (Li Yongjin et al., 2022). These findings highlight apoptosis and ferroptosis as intertwined cell death mechanisms driving IVDD pathogenesis.

#### 2.2.2 Autophagy and ER stress in IVDD

Autophagy, a self-protective mechanism for degrading damaged organelles and proteins, plays a critical role in maintaining cellular homeostasis. In IVDD, impaired autophagy disrupts this balance, leading to the accumulation of cellular waste, organelle dysfunction, and eventual cell death. Sestrins, a family of stress response proteins, promote autophagy through the mTOR pathway, reducing ER stress and protecting NP cells (Tu et al., 2018). Similarly, quercetin activates autophagy via the p38 MAPK pathway, alleviating oxidative damage in NP cells (Zhang et al., 2021).

ER stress occurs when misfolded proteins accumulate in the ER, disrupting protein balance. This stress exacerbates IVDD by inducing apoptosis and ECM degradation. For example, eicosapentaenoic acid (EPA) reduces ER stress by enhancing autophagic activity, mitigating disc degeneration (Lin et al., 2021). These findings highlight the interplay between autophagy and ER stress in IVDD, offering potential therapeutic targets to restore cellular balance and delay disease progression.

#### 2.2.3 Cellular senescence in IVDD

Aging and cellular senescence are critical intrinsic factors contributing to IVDD. As age increases, disc cells gradually exhibit senescent characteristics, such as reduced proliferative capacity and functional decline. Cellular senescence is driven by various factors, including telomere shortening, DNA damage, and genomic instability (Wang et al., 2016). Research shows that senescence-related stress induces irreversible senescence through key regulatory pathways, including p16INK4A and p53, leading to a chronic inflammatory state that exacerbates tissue degradation and drives IVDD (Vo et al., 2016). The senescence-associated secretory phenotype (SASP) factor release not only impairs nearby healthy cells but also creates a vicious cycle, further accelerating disc degeneration. Strategies aimed at delaying or reversing cellular senescence, such as telomerase activation or antioxidant application, could effectively alleviate IVDD and maintain disc structural and functional stability.

### 2.3 ECM metabolism in IVDD

The stability of the ECM, comprising collagen, proteoglycans, and glycosaminoglycans, is crucial for the structure and function of intervertebral discs. In IVDD, the balance between ECM synthesis and degradation is disrupted, leading to a significant increase in degradation rates. MMPs play an essential role in ECM degradation, with MMP-1, MMP-3, and MMP-13 notably upregulated during degeneration, directly breaking down collagen and proteoglycans and compromising the elasticity and compressive strength of the NP (Che et al., 2021). Additionally, non-coding RNAs (e.g., miR-27b and miR-133a) are involved in preventing ECM degradation by directly inhibiting MMP expression or blocking pro-inflammatory signaling, thereby preserving ECM integrity (Cazzanelli and Wuertz-Kozak, 2020). These findings suggest that regulating MMPs and related pathways could slow disc degeneration and provide new therapeutic targets for future treatments.

In addition to ECM degradation, prolonged or excessive mechanical stress can damage the structure and function of the disc. Poor posture, repetitive weight-bearing movements, and external impacts can increase the load on the disc, leading to structural damage to the AF and endplates (Vo et al., 2016). This mechanical stress not only directly damages disc structure but also triggers inflammatory responses and ECM degradation. Furthermore, mechanical stress activates signaling pathways such as TGF-β, which allows the disc to adapt to stress. However, when mechanical stress exceeds a certain threshold, TGF-β signaling becomes imbalanced, accelerating IVDD progression (Adams et al., 2015).

## 3 Mechanistic roles of TRIM proteins in IVDD

TRIM protein family is recognized for its diverse regulatory roles in cellular processes, including inflammation, cell death, autophagy, and protein metabolism. Given the multifactorial nature of IVDD, TRIM proteins emerge as critical modulators of key pathological pathways. Through their involvement in signaling networks such as NF-κB, MAPK, and β-catenin, TRIM proteins influence the balance between tissue homeostasis and degeneration.

This section delves into the mechanistic contributions of TRIM proteins to IVDD, focusing on their roles in modulating inflammation, regulating cell death pathways, and maintaining ECM metabolism. By exploring these processes, we aim to highlight how TRIM proteins serve as potential therapeutic targets in addressing IVDD progression.

### 3.1 TRIM proteins in inflammation

#### 3.1.1 General role of TRIM proteins in inflammation

The TRIM protein family is of great significance in modulating inflammatory and immune responses. As detailed in Table 1, which outlines the immunoregulatory functions of diverse TRIM proteins and their possible implications in IVDD, these proteins exert their effects through various mechanisms.

**TABLE 1:** Comprehensive summary of TRIM proteins’ immunoregulatory mechanisms and their implication in IVDD progression. The TRIM proteins are classified based on their effects on immune regulation. Positive regulators enhance inflammatory responses, negative regulators suppress them, and bidirectional regulators can either enhance or inhibit depending on the context.

TRIM protein name	Role in the immune system	Specific mechanism of action	Potential role in IVDD	References
TRIM21	Positive regulator	Augments the production of TNF - α and IL - 6 by means of the Fc receptor - mediated degradation pathway	May enhance inflammation in IVDD	Mallery et al. (2010)
TRIM25	Positive regulator	Stabilizes the IKK complex within the NF - κB signaling cascade through K63 - linked ubiquitination, enhancing the synthesis of pro - inflammatory cytokines	Could accelerate IVDD by increasing inflammation	Gack et al. (2007), Weng et al. (2014)
TRIM47	Positive regulator	Activates caspase - 3 and NF - κB - related factors, exacerbating inflammation and apoptosis	May worsen IVDD through inflammation and apoptosis	Hao et al. (2019)
TRIM38	Negative regulator	Curtails NF - κB and IRF3 activity by ubiquitinating TRAF6 and TRIF, thwarting excessive inflammatory responses	Might slow IVDD by reducing inflammation	Hu et al. (2015)
TRIM27	Negative regulator	Restrains NF - κB activity and diminishes the release of pro - inflammatory cytokines by impeding RIP1 ubiquitination	Could attenuate IVDD - related inflammation	Hatakeyama (2017b)
TRIM13	Negative regulator	Governs chronic inflammation by suppressing NF - κB activity via K48 - linked ubiquitination - mediated degradation of NEMO	May help maintain IVDD - related inflammation balance	Marzano et al. (2021)
TRIM8	Bidirectional regulator	Can both activate NF - κB signaling through TAK1 ubiquitination under specific circumstances and deubiquitinate certain NF - κB signaling molecules to attenuate the inflammatory response	May have variable effects on IVDD inflammation	Liu et al. (2020); Bai et al. (2020)
TRIM30	Bidirectional regulator	Capable of either enhancing or inhibiting pro - inflammatory responses via distinct mechanisms within the NF - κB pathway	Could impact IVDD inflammation either way	Deng et al. (2022)
TRIM33	Bidirectional regulator	Modulates the functions of TRAF3 and NEMO, augmenting or suppressing macrophage inflammatory responses depending on the infection status	May regulate IVDD inflammation context - dependently	Roy and Singh (2021)

TRIM, Tripartite Motif - containing protein family; IVDD, intervertebral disc degeneration; NF - κB, Nuclear Factor kappa - light - chain - enhancer of activated B cells; IKK, IκB kinase; TNF - α, Tumor Necrosis Factor - α; IL, 6, Interleukin - 6; IRF3, Interferon Regulatory Factor 3; TRAF6, TNF, Receptor - Associated Factor 6; TRIF, TIR, Domain - Containing Adapter - Inducing Interferon - β; NEMO, NF - κB, essential modulator.

##### 3.1.1.1 Positive regulators

Certain TRIM proteins function as positive regulators in the inflammatory context. TRIM21 augments the production of TNF - α and IL - 6 by means of the Fc receptor - mediated degradation pathway, thereby bolstering local immune defenses (Mallery et al., 2010). TRIM25 stabilizes the IKK complex within the NF - κB signaling cascade through K63 - linked ubiquitination, consequently enhancing the synthesis of pro - inflammatory cytokines (Gack et al., 2007; Weng et al., 2014). In the case of ischemia - reperfusion injury, TRIM47 activates caspase - 3 and NF - κB - related factors, exacerbating inflammation and apoptosis (Hao et al., 2019).

##### 3.1.1.2 Negative regulators

Another subset of TRIM proteins acts as negative regulators. TRIM38 curtails NF - κB and IRF3 activity by ubiquitinating TRAF6 and TRIF, effectively thwarting excessive inflammatory responses (Hu et al., 2015). TRIM27 restrains NF - κB activity and diminishes the release of pro - inflammatory cytokines by impeding RIP1 ubiquitination (Hatakeyama, 2017b). TRIM13 governs chronic inflammation by suppressing NF - κB activity via K48 - linked ubiquitination - mediated degradation of NEMO (Marzano et al., 2021).

##### 3.1.1.3 Bidirectional regulatory functions

Some TRIM proteins possess bidirectional regulatory capabilities. TRIM8 can both activate NF - κB signaling through TAK1 ubiquitination under specific circumstances and deubiquitinate certain NF - κB signaling molecules to attenuate the inflammatory response (Liu et al., 2020; Bai et al., 2020). TRIM30 is capable of either enhancing or inhibiting pro - inflammatory responses via distinct mechanisms within the NF - κB pathway (Deng et al., 2022). TRIM33 modulates the functions of TRAF3 and NEMO, augmenting or suppressing macrophage inflammatory responses depending on the infection status (Roy and Singh, 2021).

Overall, TRIM proteins predominantly regulate inflammation through ubiquitination - related mechanisms that modify key molecules in inflammatory signaling pathways. Their multifaceted regulatory functions, including bidirectional regulation, contribute to the intricate network that maintains immune homeostasis.

#### 3.1.2 Contribution of TRIM proteins to IVDD via inflammation

Inflammation represents a central pathological process in IVDD, and members of the TRIM protein family play pivotal roles in modulating pro-inflammatory signals and cytokine expression within disc cells. By activating inflammatory pathways such as NF - κB and MAPK, TRIM proteins markedly exacerbate the inflammatory response in both NP and AF cells, thereby propelling the progression of IVDD.

In the context of the NF - κB pathway, upon activation by TNF - α or other inflammatory factors, TRIM14 further augments the activity of the NF - κB pathway. The TRIM14 - mediated activation of NF - κB not only leads to a substantial increase in the production of pro-inflammatory factors, including TNF - α and IL - 1β, in NP cells but also upregulates the expression of MMPs, consequently accelerating ECM degradation (Zhu et al., 2019). This mechanism intensifies the inflammatory milieu in NP and AF cells, exacerbates the local inflammatory environment, and ultimately destabilizes the disc structure.

Simultaneously, TRIM56 plays a crucial part in MAPK - dependent inflammatory senescence. Research findings indicate that TRIM56 ubiquitinates ATR (Ataxia Telangiectasia and Rad3 - Related Protein), thereby triggering the DNA damage response and activating the MAPK pathway, which drives NP cells into a state of inflammatory senescence. Additionally, the cGAS - STING pathway, a vital pathway for DNA damage and inflammatory responses, is activated by TRIM56, leading to inflammatory senescence in NP cells and aggravating IVDD (Zhang et al., 2024). Therefore, the abnormal upregulation of TRIM proteins not only deteriorates the local inflammatory response but also induces the transition of disc cells into a senescent state, thus fueling the advancement of IVDD.

### 3.2 TRIM proteins in cell death

#### 3.2.1 Regulation of cell death by TRIM proteins

The TRIM protein family is intricately involved in several fundamental cellular processes that are essential for maintaining genomic integrity, controlling cell fate, and ensuring proper tissue homeostasis. These processes include DNA repair, apoptosis, and cell differentiation, each of which is regulated by different members of the TRIM family in distinct yet interconnected ways. The relevant content is summarized in Table 2.

**TABLE 2:** Summary of the roles of TRIM proteins in cell death regulation related to IVDD. The TRIM proteins are grouped based on their known functions in cell death regulation processes, including DNA repair, apoptosis, and cell differentiation. These processes are interconnected and play crucial roles in maintaining cell homeostasis and tissue integrity. Dysregulation of these processes can have significant implications for IVDD development.

TRIM protein name	Role in cell death regulation	Specific mechanism of action	Potential role in IVDD	References
TRIM29	Involved in DNA repair	Acts as a scaffold, facilitating the assembly of DNA repair complexes like BRCA1 complex and DNA - PKcs on chromatin. Stabilizes the DDR machinery at DNA double - strand breaks (DSBs) by interacting with modified histones	May maintain genomic stability, affect cell survival and IVDD progression. Impaired DNA repair could lead to increased cell death and disc degeneration	Masuda et al. (2015)
TRIM28	Involved in DNA repair and apoptosis	As an E3 ubiquitin ligase, phosphorylated by ATM kinase under DNA damage conditions. Triggers the ubiquitination and degradation of SIRT1. Interacts with 53BP1 to support Non - Homologous End Joining (NHEJ) repair	Plays a role in DNA damage response and apoptosis. Dysregulation may disrupt cell survival - death balance, influencing IVDD	McAvera and Lisa (2020), Ouyang et al. (2022)
TRIM52	Regulates apoptosis	Upregulates the activity of IKKβ and P65 within the NF - κB signaling pathway	May affect cell survival and apoptosis. Abnormal activation could disrupt normal cell death, contributing to IVDD	Bai and Tang (2023), Yang et al. (2018)
TRIM14	Regulates apoptosis and activates AKT signaling	Activates the NF - κB pathway when overexpressed, promoting cell proliferation and resistance to apoptosis. Also activates the AKT signaling pathway	Can influence apoptotic and proliferative behavior. Dysregulation may lead to abnormal cell growth and death, affecting disc structure and function in IVDD	Jiang et al. (2020), Xu et al. (2017)
TRIM46	Regulates apoptosis	Activates NF - κB signaling through the ubiquitination of PPARα	Plays a role in apoptosis regulation. Imbalance could disrupt apoptotic balance, contributing to IVDD progression	Jiang et al. (2020)
TRIM24	Regulates apoptosis and DNA damage repair	Inhibits the efficiency of DNA damage repair by ubiquitinating and suppressing the activity of p53 and its downstream DDR genes. Modulates the NF - κB and p53 signaling pathways	Affects DNA repair and apoptosis. Dysfunction may lead to increased cell death and genomic instability, promoting IVDD	Liu et al. (2021), Bai and Tang (2023)
TRIM16	Induces apoptosis	Directly activates caspase - 2. Prolongs the half - life of the TDP43 protein and suppresses the activities of cell cycle proteins pRb and E2F1	Can induce apoptosis. Altered expression may disrupt normal cell cycle and apoptotic processes, potentially involved in IVDD	Liu et al. (2021), Bai and Tang (2023)
TRIM32	Regulates apoptosis and cell differentiation	Promotes the polyubiquitination and degradation of cMyc, inhibiting neural stem cell proliferation and steering their differentiation towards the nervous system	May affect cell fate and survival. Aberrant regulation could contribute to IVDD	Liu et al. (2021), Bahnassawy et al. (2015)
TRIM71	Regulates cell differentiation	Binds to specific mRNAs and inhibits their expression. Regulates the miRNA let - 7 through dual mechanisms	Plays a role in maintaining stem cell properties. Dysregulation could impact cell differentiation and potentially be related to IVDD	Mitschka et al. (2015), Connacher and Goldstrohm (2021), Fernández et al. (2021)

DDR, DNA, damage response; BRCA1, Breast Cancer Type 1 Susceptibility Protein; DNA - PKcs, DNA, Dependent Protein Kinase Catalytic Subunit; H3K36me2/3, Histone H3 Lysine 36 Dimethylation/Trimethylation; H4K16Ac, Histone H4 Lysine 16 Acetylation; DSB, DNA, Double - Strand Break; TIF1, Transcriptional Intermediary Factor 1; NHEJ, Non - Homologous End Joining; HR, homologous recombination; PARP1, Poly - ADP, Ribose Polymerase 1; NF - κB, Nuclear Factor kappa - light - chain - enhancer of activated B cells; p53, Tumor protein p53; Oct4, Octamer - binding Transcription Factor 4; cMyc, Cellular Myelocytomatosis; miRNA, MicroRNA; LIN28, LIN28 Homolog A; TUT4, Terminal Uridylyl Transferase 4; AGO2, Argonaute 2; RISC, RNA, Induced Silencing Complex; iPSC, induced pluripotent stem cell; PHD, plant homeodomain; AKT, Protein Kinase B; TDP43, TAR DNA, Binding Protein 43; pRb, Retinoblastoma Protein; E2F1, E2F Transcription Factor 1.

##### 3.2.1.1 DNA repair

TRIM29 is a key player in the DNA Damage Response (DDR). It acts as a scaffold, facilitating the assembly of crucial DNA repair complexes such as the BRCA1 complex and DNA - PKcs on chromatin. By interacting with modified histones, specifically H3K36me2/3 and H4K16Ac, TRIM29 stabilizes the DDR machinery at sites of DNA double - strand breaks (DSBs). This stabilization activates the DDR cascade and enhances the cell’s ability to cope with DNA damage, thereby safeguarding genomic stability (Masuda et al., 2015).

The TIF1 family member, TRIM28, also has a significant role in DNA repair. Functioning as an E3 ubiquitin ligase, TRIM28 is phosphorylated by ATM kinase under DNA damage conditions. This phosphorylation event triggers the ubiquitination and subsequent degradation of SIRT1, a protein that otherwise modulates the DNA damage response. By limiting SIRT1’s activity, TRIM28 further augments the effectiveness of the DDR. Additionally, TRIM28 physically interacts with 53BP1, a key factor in Non - Homologous End Joining (NHEJ) repair, to support this repair pathway (McAvera and Lisa, 2020; Ouyang et al., 2022).

##### 3.2.1.2 Apoptosis

TRIM52 has been shown to upregulate the activity of IKKβ and P65 within the NF - κB signaling pathway, especially in ovarian cancer. This activation leads to enhanced cell survival by suppressing apoptosis, allowing cancer cells to evade normal cell death mechanisms and potentially contribute to tumor progression (Bai and Tang, 2023; Yang et al., 2018).

Meanwhile, osteosarcoma cells are affected by TRIM14 and TRIM46 in the context of apoptosis regulation. TRIM14, when overexpressed, activates the NF - κB pathway, which in turn promotes cell proliferation and confers resistance to apoptosis. Interestingly, the targeted inhibition of TRIM14 using the Chanti - TRIM antibody has been demonstrated to reduce NF - κB activity and induce apoptosis in these cells (Madhu et al., 2021). TRIM46, on the other hand, activates NF - κB signaling through the ubiquitination of PPARα. This activation promotes osteosarcoma cell proliferation and anti - apoptotic behavior. Conversely, inhibiting TRIM46 effectively attenuates NF - κB pathway activity, leading to the induction of apoptosis in cancer cells (Jiang et al., 2020).

TRIM24 also plays a dual role in apoptosis regulation. It inhibits the efficiency of DNA damage repair by ubiquitinating and suppressing the activity of p53 and its downstream DDR genes. Additionally, TRIM24 modulates both the NF - κB and p53 signaling pathways to influence apoptosis. Meanwhile, TRIM16 uniquely induces apoptosis in breast cancer cells by directly activating caspase - 2. TRIM16 also prolongs the half - life of the TDP43 protein and suppresses the activities of cell cycle proteins pRb and E2F1, all of which contribute to an increased propensity for apoptosis (Liu et al., 2021; Bai and Tang, 2023).

##### 3.2.1.3 Cell differentiation

TRIM32 is involved in the regulation of stem cell differentiation. It modulates the stability of pluripotency factors, most notably Oct4 and cMyc. Specifically, TRIM32 promotes the polyubiquitination and subsequent degradation of cMyc. This degradation event inhibits the proliferation of neural stem cells and steers their differentiation towards the nervous system. Notably, studies have shown that mouse embryonic fibroblasts lacking TRIM32 exhibit enhanced reprogramming ability, emphasizing TRIM32’s crucial role in maintaining specific cell fates and suppressing inappropriate cellular reprogramming (Liu et al., 2021; Bahnassawy et al., 2015).

In neural and germline stem cells, the TRIM - NHL family member, TRIM71, is essential for proper differentiation. TRIM71 binds to specific mRNAs and inhibits their expression. This binding activity ensures the precise temporal and spatial expression of key genes required for differentiation, thereby preventing premature differentiation and maintaining the stem cells in an undifferentiated, proliferative state. Additionally, TRIM71 regulates the miRNA let - 7 through a dual mechanism. It interacts with LIN28 and TUT4 to prevent let - 7 maturation and also binds to AGO2 within the RISC complex to suppress let - 7 activity. These regulatory actions of TRIM71 are crucial for maintaining stem cell proliferation and the undifferentiated state (Mitschka et al., 2015; Connacher and Goldstrohm, 2021; Fernández et al., 2021).

TRIM28 also contributes to the maintenance of stem cell pluripotency. The RING and PHD domains of TRIM28 are indispensable for the pluripotency and self - renewal of induced pluripotent stem cells (iPSCs). Loss of these domains leads to significant induction of stem cell differentiation, highlighting TRIM28’s central role in regulating stem cell self - renewal and pluripotency (Mazurek et al., 2021).

Some TRIM family members, such as TRIM14 in osteosarcoma cells, can influence cell behavior by activating specific signaling pathways. TRIM14 activates the AKT signaling pathway, which in turn enhances cell proliferation and invasiveness. This activation provides a mechanistic explanation for TRIM14’s role in osteosarcoma cells (Xu et al., 2017).

In conclusion, the TRIM protein family exerts a complex and multifaceted influence on DNA repair, apoptosis, and cell differentiation. The proper regulation of these processes by TRIM proteins is crucial for maintaining genomic stability, cell survival, and the correct development and function of tissues. Dysregulation of these processes can have far - reaching implications in various diseases, including potential contributions to the progression of IVDD, as summarized in Table 2. Understanding the detailed mechanisms by which the TRIM protein family regulates these cellular processes not only provides insights into fundamental cell biology but also holds great promise for the development of novel therapeutic strategies.

#### 3.2.2 Contribution of TRIM proteins to IVDD via cell death

Apoptosis is a critical pathological process in IVDD, particularly affecting NP cells. It significantly influences the structure and function of the intervertebral disc. Various members of the TRIM protein family regulate apoptosis pathways, modulating the sensitivity of NP cells to inflammation and oxidative stress through endogenous and exogenous apoptotic signals.

In terms of endogenous apoptotic signaling, TRIM32 plays a notable role. Research shows that TRIM32 ubiquitinates Axin1, activating the β-catenin signaling pathway and subsequently increasing NP cell apoptosis rates. When NP cells are exposed to inflammatory factors such as TNF-α, TRIM32 expression is upregulated. This upregulation leads to TRIM32-mediated ubiquitination of Axin1, disrupting β-catenin homeostasis, triggering endogenous apoptotic signaling, and accelerating IVDD progression (Chen et al., 2020). Interestingly, studies have also demonstrated that exosomes derived from bone marrow mesenchymal stem cells can inhibit TRIM32 expression by delivering miR-155-5p, effectively reducing TNF-α-induced NP cell apoptosis (Chen et al., 2024). These findings suggest that targeting TRIM32 or enhancing miR-155-5p expression could be potential therapeutic strategies to mitigate IVDD.

In addition, TRIM21 contributes to apoptosis in NP cells via exogenous pathways. The hypoxic environment surrounding NP cells relies heavily on HIF-1α (Hypoxia-Inducible Factor 1-α) to maintain metabolic balance and hypoxic survival. However, under oxidative stress conditions, TRIM21 overexpression accelerates HIF-1α ubiquitination and degradation as an E3 ubiquitin ligase. This degradation deprives NP cells of hypoxic protection, advancing IVDD progression (Zheng et al., 2021). Furthermore, TRIM14, under TNF-α stimulation, activates the NF-κB pathway, increasing the expression of the pro-apoptotic protein Bax while inhibiting the anti-apoptotic protein Bcl-2. This shift heightens NP cells’ sensitivity to inflammatory stress and contributes to disc structural instability (Zhu et al., 2019). Thus, the upregulation of TRIM14 and TRIM21 exacerbates apoptosis and undermines the integrity of intervertebral discs.

Apart from apoptosis, autophagy and endoplasmic reticulum (ER) stress also play significant roles in IVDD pathogenesis. These processes are critical cellular mechanisms that respond to stress by maintaining homeostasis. The regulatory roles of TRIM proteins in autophagy and ER stress offer new insights into slowing IVDD progression.

Under oxidative stress conditions, TRIM21 interacts with autophagy-related proteins Beclin1 and LC3, enhancing the autophagic capacity of NP cells. This process aids in clearing damaged mitochondria, alleviating oxidative stress, and delaying the progression of IVDD [80]. Additionally, TRIM28 plays a protective role by reducing ER stress-induced apoptosis. It prevents the accumulation of misfolded proteins, thereby maintaining cellular homeostasis and supporting NP cell survival (Chen et al., 2023).

In summary, the regulation of autophagy and ER stress by TRIM proteins improves the stress resilience of disc cells, particularly NP and annulus fibrosus (AF) cells. These mechanisms provide a protective role in IVDD. However, it is worth noting that excessive autophagy under certain conditions might adversely affect cellular viability, highlighting the need for a balanced regulatory approach.

### 3.3 TRIM proteins in ECM metabolism

#### 3.3.1 Regulation of protein metabolism by TRIM proteins

The TRIM protein family is deeply involved in maintaining protein homeostasis by regulating protein degradation through mechanisms such as selective autophagy, ubiquitination, and the proteasome system. These processes are essential for addressing cellular challenges, including protein misfolding, endoplasmic reticulum (ER) stress, and cell membrane damage. Recent studies have revealed diverse roles of individual TRIM proteins in maintaining cellular protein quality, shedding light on their physiological and pathological significance (Lei et al., 2021).

One prominent example is TRIM32, which regulates selective autophagy by mono-ubiquitinating the autophagy receptor p62/SQSTM1 (Sequestosome 1). This interaction facilitates the formation of punctate structures that selectively target damaged or misfolded proteins for autophagic degradation, thus ensuring cellular protein quality control. However, mutations in TRIM32, such as those associated with limb-girdle muscular dystrophy type 2H (LGMD2H), impair its ability to activate p62. This results in the accumulation of harmful proteins and increased cellular damage, particularly in muscle cells, highlighting its critical role in protecting against cellular stress (Overå et al., 2019; Di et al., 2020).

In addition to TRIM32, TRIM13 has been shown to play a key role in mitigating ER stress. By initiating autophagy through its coiled-coil domain, TRIM13 facilitates the clearance of misfolded proteins within the ER, alleviating stress-induced damage and supporting cell survival. This mechanism provides an essential adaptive response to metabolic and environmental stress (Hatakeyama, 2017a). Similarly, TRIM16 contributes to cellular resilience by identifying and responding to membrane damage. It interacts with autophagy-related proteins ULK1 and Beclin 1 to regulate lysosomal quality control and enhance the cell’s ability to manage membrane integrity under pathogenic or stress conditions (Kumar et al., 2017).

In cardiac muscle cells, TRIM55 (MuRF2) and TRIM63 (MuRF1) are vital for maintaining protein homeostasis via the proteasomal degradation pathway. These proteins target cardiac-specific proteins, such as troponin I, for degradation, thereby preventing cardiac hypertrophy and reducing oxidative stress. By protecting against heart failure, TRIM55 and TRIM63 underscore the importance of protein turnover in preserving cardiac function under pathological conditions (Zhang et al., 2020).

Another notable member, TRIM72 (MG53), is indispensable for cell membrane repair, particularly during ischemia/reperfusion injury. It binds to phospholipids at sites of membrane damage, promoting rapid repair and activating the PI3K-Akt-GSK3β and ERK1/2 signaling pathways to enhance cardioprotection. Moreover, TRIM72 plays a role in metabolic regulation by modulating insulin receptor degradation in diabetic cardiomyopathy, which affects insulin sensitivity and presents a potential therapeutic target in cardiovascular diseases (Zhang et al., 2020).

Taken together, TRIM proteins are pivotal regulators of protein homeostasis across diverse cellular contexts. Their roles in autophagy, ubiquitination, and proteasomal degradation provide critical protective and adaptive mechanisms, particularly under conditions of cellular stress or pathology. Future research focusing on the molecular mechanisms of TRIM proteins in disease-specific contexts could unlock novel therapeutic strategies for managing muscular, cardiovascular, and metabolic disorders (Beese et al., 2019; Mandell et al., 2014). The detailed data in Table 3 systematically illustrates the functions of TRIM proteins in protein metabolism, highlighting their mechanisms and potential relevance to IVDD.

**TABLE 3:** Summary of the roles of TRIM proteins in protein metabolism and their implication in IVDD.

TRIM protein name	Specific mechanism of action	Potential role in IVDD	References
TRIM32	Mono - ubiquitinates p62/SQSTM1 to target damaged/misfolded proteins for autophagic degradation	Maintains protein quality; impairment may cause protein accumulation and cell damage, contributing to IVDD	Overå et al. (2019), Di et al. (2020)
TRIM13	Initiates autophagy via coiled - coil domain to clear ER misfolded proteins	Alleviates ER stress - related damage; dysfunction may disrupt homeostasis and affect IVDD	Hatakeyama (2017a)
TRIM16	Interacts with ULK1 and Beclin 1 to regulate lysosomal quality control	Maintains membrane integrity; altered expression may impact cell viability and IVDD	Kumar et al. (2017)
TRIM55 & TRIM63	Target cardiac proteins like troponin I for proteasomal degradation	May influence disc cell protein turnover/function if conserved; dysregulation could affect disc health	Zhang et al. (2020a)
TRIM72	Binds phospholipids for membrane repair and modulates insulin receptor degradation	Affects cell membrane integrity/metabolism; abnormalities may contribute to IVDD	Zhang et al. (2020b)

ER, endoplasmic reticulum; ULK1, Unc - 51, like kinase 1.

#### 3.3.2 Contribution of TRIM proteins to ECM metabolism in IVDD

The stability of the ECM is essential for the mechanical function of the intervertebral disc. Imbalance in ECM degradation is a hallmark of IVDD, with TRIM proteins influencing disc stability by modulating ECM metabolism-related molecules and pathways. TRIM16 and TRIM32 play important roles in ECM degradation, particularly by regulating the expression of MMPs, which impacts the structural integrity of NP and AF cells.

In the inflammatory environment of IVDD, TRIM16 promotes IL-1β secretion through a process called secretory autophagy, a specialized autophagic pathway involved in releasing cellular components. This secretion subsequently activates MMPs, leading to accelerated ECM degradation. This mechanism weakens the structural support of NP and AF cells, diminishing the mechanical properties of the disc and making it more prone to degeneration (Zheng et al., 2021). Furthermore, TRIM32 upregulates the β-catenin signaling pathway, thereby increasing MMP expression and further driving ECM degradation. The β-catenin pathway, which plays a critical role in cell proliferation and differentiation, also significantly influences ECM synthesis and degradation. TRIM32’s regulatory role within this pathway makes NP and AF cells more susceptible to matrix breakdown under inflammatory conditions, leading to a loosening of disc structure (Chen et al., 2020). By targeting the effects of TRIM proteins on MMPs, it may be possible to delay disc degeneration and improve disc functionality in IVDD.

## 4 Summary and future directions

The summarized data in Tables 4, 5 highlight the multifaceted roles of TRIM proteins in IVDD pathogenesis, including their involvement in ECM degradation, inflammatory signaling, and apoptotic pathways. These findings underscore their potential as therapeutic targets in mitigating disc degeneration.

**TABLE 4:** Roles of TRIM proteins in IVDD pathogenesis.

TRIM protein	Key roles in IVDD	Underlying mechanism	Citations
TRIM16	Promotes ECM degradation	Promotes IL-1β secretion via secretory autophagy, activates MMPs, weakens structural support of NP and AF cells	Zheng et al. (2021a)
TRIM32	Promotes apoptosis and ECM degradation	Ubiquitinates Axin1, activates β-catenin signaling pathway; regulates ECM synthesis and degradation	Chen et al., (2020a), Chen et al. (2024b)
TRIM21	Accelerates apoptosis, enhances autophagy	Acts as an E3 ubiquitin ligase, promotes ubiquitination and degradation of HIF-1α; interacts with Beclin1 and LC3 to enhance autophagy	Zheng et al. (2021b), Chen et al. (2023)
TRIM14	Promotes inflammation and apoptosis	Activates the NF-κB signaling pathway, increases Bax expression, inhibits Bcl-2 expression	Zhu et al. (2019)
TRIM56	Drives inflammatory senescence	Ubiquitinates ATR, activates MAPK pathway, drives inflammatory senescence; activates the cGAS-STING pathway	Zhang et al. (2024)

IVDD, intervertebral disc degeneration; ECM, extracellular matrix; MMPs, Matrix Metalloproteinases; NP, nucleus pulposus; AF, annulus fibrosus; NF-κB, Nuclear Factor Kappa-B; HIF-1α, Hypoxia-Inducible Factor 1-Alpha; MAPK, Mitogen-Activated Protein Kinase; ATR, Ataxia Telangiectasia and Rad3-Related Protein; cGAS, Cyclic GMP-AMP, synthase; STING, stimulator of interferon genes.

**TABLE 5:** Regulatory roles of TRIM proteins in cellular signaling pathways associated with IVDD.

TRIM protein	Pathway	Pathway status	Mechanism	References
TRIM21	NF-κB signaling pathway	Activation	Catalyzes K63-linked ubiquitination of the IKK complex, stabilizing NF-κB signaling and promoting the expression of TNF-α and IL-6	Gack et al. (2007), Mallery et al. (2010)
TRIM38	NF-κB/IRF3 signaling pathway	Inhibition	Ubiquitinates TRAF6 and TRIF, leading to the suppression of pro-inflammatory cytokine production and maintaining immune homeostasis	Hu et al. (2015)
TRIM32	Wnt/β-catenin signaling pathway	Activation	Ubiquitinates AXIN1, disrupting β-catenin degradation and amplifying signaling, which promotes NP cell apoptosis under inflammatory conditions	Chen et al. (2020b), Chen et al. (2024a)
TRIM56	MAPK signaling pathway	Activation	Facilitates the ubiquitination of ATR, triggering the DNA damage response and activating MAPK signaling, thereby driving inflammatory senescence	Zhang et al. (2024)
TRIM14	NF-κB signaling pathway	Activation	Enhances NF-κB activity under TNF-α stimulation, increasing Bax expression and suppressing Bcl-2, which induces apoptosis in NP cells	Zhu, Sun, and Shen (2019)
TRIM29	DNA damage repair pathway	Activation	Stabilizes DDR complexes (e.g., BRCA1) at sites of DNA double-strand breaks, improving repair efficiency	Masuda et al. (2015)
TRIM28	DNA repair/NHEJ pathway	Activation	NHEJ repair by ubiquitinating SIRT1, which enhances the recruitment of repair factors	Ouyang et al. (2022)
TRIM13	ER Stress	Activation	Induces autophagy to clear misfolded proteins, alleviating ER stress and maintaining cellular homeostasis	Hatakeyama (2017a)
TRIM16	IL-1β signaling pathway/ECM metabolism	Activation	Facilitates IL-1β secretion via secretory autophagy, which indirectly activates MMPs and accelerates ECM degradation in intervertebral discs	Zheng et al. (2021a)
TRIM72	PI3K-Akt signaling pathway	Activation	Localizes to membrane injury sites, binding to phospholipids and activating the PI3K-Akt pathway to promote membrane repair and cell survival	Zhang et al. (2020a)

NF-κB, Nuclear Factor kappa-light-chain-enhancer of activated B cells; IRF3, Interferon Regulatory Factor 3; MAPK, Mitogen-Activated Protein Kinase; DDR, DNA, Damage Response; NHEJ, Non-Homologous End Joining; ER, Stress, Endoplasmic Reticulum Stress; PI3K-Akt, Phosphoinositide 3-Kinase/Protein Kinase B; ECM, Extracellular Matrix; MMPs, Matrix Metalloproteinases; TNF-α, Tumor Necrosis Factor-alpha; IL-1β, Interleukin-1, beta; Bax, Bcl-2-associated X protein; Bcl-2, B-cell lymphoma 2; AXIN1, Axis Inhibitor 1; ATR, Ataxia Telangiectasia and Rad3-related protein; BRCA1, Breast Cancer 1.

### 4.1 Challenges in translational research

The translational application of TRIM protein-based therapies in intervertebral disc degeneration (IVDD) is associated with several critical challenges. These challenges, including drug delivery methods, specificity of modulation, and safety concerns, must be systematically addressed to advance clinical translation.

#### 4.1.1 Drug delivery methods

Effective drug delivery remains a significant hurdle in targeting TRIM proteins for IVDD treatment. The complex anatomy of the intervertebral disc, characterized by its avascular nature and dense extracellular matrix, poses significant barriers to therapeutic penetration. Systemic delivery methods lack specificity, reducing efficacy and increasing off-target risks. Advanced approaches, such as nanoparticle systems or intradiscal injections, may enhance site-specific delivery and ensure sustained effects (Henry et al., 2018; Pan et al., 2018; Chen et al., 2020).

#### 4.1.2 Specificity of TRIM protein modulation

TRIM proteins exhibit multifunctionality, regulating diverse cellular pathways that vary across tissues. This functional complexity raises concerns regarding unintended off-target effects when modulating TRIM proteins. For instance, TRIM32 affects both apoptosis and ECM metabolism, creating a risk of non-specific cellular responses (Chen et al., 2020). Precision medicine approaches such as RNA interference (siRNA) or CRISPR-Cas9-mediated gene editing offer potential strategies to enhance specificity. Additionally, identifying tissue- or cell-specific biomarkers could facilitate selective targeting, ensuring that TRIM protein modulation is restricted to pathological environments within the disc (DiStefano et al., 2022a).

#### 4.1.3 Safety concerns

Safety remains a paramount concern in developing TRIM protein-based therapies. Modulating key regulatory proteins could disrupt cellular homeostasis and trigger adverse reactions, including immune responses or cytotoxicity (Matta and Mark Erwin, 2020). Rigorous preclinical studies using *in vitro* and *in vivo* models are necessary to evaluate long-term safety and efficacy. Furthermore, clinical trials with stratified patient cohorts could help identify patient-specific risks, paving the way for safer therapeutic protocols (Panebianco et al., 2020).

In summary, overcoming these challenges requires a multidisciplinary approach, combining advanced drug delivery technologies, precision targeting strategies, and comprehensive safety evaluations. Addressing these issues will be essential for translating TRIM protein-based therapies into effective clinical treatments for IVDD.

### 4.2 Potential for synergistic therapies

Integrating TRIM protein modulation with other therapeutic approaches, such as anti-inflammatory drugs and stem cell-based therapies, can enhance treatment efficacy and better address the multifactorial pathology of IVDD.

#### 4.2.1 Combination with anti-inflammatory agents

Inflammation is a key pathological driver of IVDD, contributing to ECM degradation and cell death. TRIM proteins, such as TRIM14 and TRIM21, regulate inflammation through NF-κB and MAPK pathways. Combining TRIM protein-targeted therapies with anti-inflammatory agents, such as TNF-α or IL-1β inhibitors, could significantly alleviate the inflammatory environment and preserve disc structure (DiStefano et al., 2022b). Additionally, such combinations may optimize dosing strategies, reducing drug toxicity while maintaining efficacy.

#### 4.2.2 Integration with stem cell therapies

Stem cell-based therapies, particularly MSCs, hold great potential for ECM repair and cell survival. The regulatory role of TRIM proteins in ECM metabolism and apoptosis can synergize with stem cell therapies. For instance, inhibiting TRIM32 has been shown to enhance the functions of MSC-derived exosomes, reducing inflammation and promoting ECM regeneration (Wang et al., 2015). Furthermore, MSCs have demonstrated the ability to restore disc height and regenerate the extracellular matrix in animal models of IVDD (Li et al., 2022).

#### 4.2.3 Future research directions

1. Optimizing Therapeutic Combinations: Investigate optimal combinations of TRIM protein modulation with anti-inflammatory drugs or stem cell-based therapies to achieve synergistic effects (Ohnishi et al., 2023).
2. Personalized Treatment: Develop targeted treatment protocols based on patient-specific biomarkers to enhance precision and efficacy (Tong et al., 2017).
3. Clinical Validation: Conduct large-scale trials to validate the safety and efficacy of combination therapies, facilitating their translation into clinical practice (Masuda, 2008).

In summary, addressing challenges such as drug delivery, specificity, and safety is critical for advancing TRIM protein therapies in IVDD. Simultaneously, integrating TRIM protein modulation with anti-inflammatory agents and stem cell therapies offers synergistic potential to tackle the multifactorial pathology of IVDD. Future efforts should focus on optimizing therapeutic combinations, personalizing treatment strategies, and conducting robust clinical trials to translate these therapies into effective clinical solutions.
